# Partners’ engagement in surveillance among survivors of colorectal cancer: A qualitative study

**DOI:** 10.1002/cam4.3725

**Published:** 2021-01-18

**Authors:** Christine M. Veenstra, Jennifer Acosta, Rebecca Sharar, Sarah T. Hawley, Arden M. Morris

**Affiliations:** ^1^ Division of Hematology/Oncology Department of Internal Medicine University of Michigan Ann Arbor Michigan USA; ^2^ Institute for Healthcare Policy and Innovation University of Michigan Ann Arbor Michigan USA; ^3^ Johns Hopkins School of Medicine Baltimore Maryland USA; ^4^ University of Washington School of Medicine Seattle Washington USA; ^5^ Department of Internal Medicine University of Michigan Ann Arbor Michigan USA; ^6^ Department of Surgery S‐Spire Center Stanford University Stanford California USA

**Keywords:** cancer, colorectal cancer, dyadic, oncology, partners, qualitative, spouses, surveillance, survivorship

## Abstract

**Objectives:**

Following treatment of Stage III colorectal cancer, guidelines recommend 3–5 years of surveillance for recurrence. However, over half of the 1.2 million U.S. survivors of colorectal cancer fail to receive guideline‐concordant surveillance. In light of growing recognition that members of couples are interdependent and influence each other's health behaviors, we sought to describe, in their own words, the perspectives of spouses/partners on their engagement in patients’ surveillance.

**Methods:**

We conducted in‐person, semi‐structured interviews with 10 survivors of stage III colorectal cancer and their partners, together and separately. Interviews were transcribed verbatim, iteratively coded, and analyzed to identify emergent themes pertaining to partner engagement. Findings were validated through triangulation between study team members and triangulation between dyadic and individual interviews, and through an extensive search of transcripts for disconfirming evidence.

**Results:**

We identified three overarching domains of partner engagement in surveillance. First, *psychosocial engagement* included promoting patient participation in surveillance, showing care and concern, and attending to partner self‐care. Second, *information‐seeking and dyadic communication* focused on gathering information, staying informed about test results, and communicating about surveillance. Third, *instrumental engagement* referred to any explicit, objective activities such as scheduling appointments, attending appointments, and managing responsibilities at home. Participants shared strategies, examples, and in some cases unmet needs.

**Conclusions:**

This study generated new, clinically meaningful knowledge about the ways in which partners engage in patients’ surveillance. Opportunities to leverage partners as informal resources in surveillance include development of dyadic interventions to help partners engage most effectively.

## Graphical abstract

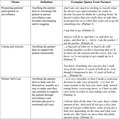

## 1 BACKGROUND

After completion of curative‐intent treatment of Stage III colorectal cancer, over 40% of patients will experience a cancer recurrence.^1^ Evidence‐based guidelines recommend a regimen of surveillance among survivors to detect potential recurrence prior to the development of symptoms, while there is still an opportunity for surgical cure.^2^ Guideline‐concordant surveillance includes a combination of physician visits, laboratory studies, imaging studies, and colonoscopy for a period of 3–5 years.^2^, ^3^, ^4^ It is important to recognize, however, that this regimen may be burdensome for some individuals. Indeed, over half of the 1.2 million colorectal cancer survivors in the United States fail to receive guideline‐concordant surveillance.^5^, ^6^ This failure to receive recommended care may represent a missed opportunity to detect and cure limited cancer recurrences.

The gap between ideal and actual care in the 50% of survivors who do not receive optimal surveillance mandates inquiry into patient and family perspectives of surveillance. Understanding the role of informal support systems, that is, the spouses/partners of patients, and then, leveraging those informal support systems, may provide an opportunity to improve quality of care and meaningful clinical outcomes among survivors of colorectal cancer. Approximately 60% of colorectal cancer patients are married or partnered.^7^ Engaging the partners of patients, who belong to, according to the National Cancer Institute, “part of the survivorship experience,” may represent one approach to improving cancer surveillance. It has been shown that patients’ and partners’ responses to cancer are interdependent and influence each other's attitudes, health behaviors, and health outcomes.^8^ Previous work has shown that partners participate in and contribute to decision‐making around patients’ options for cancer treatment,^9^, ^10^ and that partners sometimes value aggressive cancer screening and treatment options more than patients do themselves.^11^ It is likely that a partner's involvement extends across the continuum of cancer care into the survivorship period, although this has not been studied and virtually nothing is known about the role that partners play in patients’ uptake of surveillance following the transition to survivorship.

Addressing this gap in understanding the engagement of partners in surveillance may inform dyadic interventions to increase uptake of guideline‐concordant surveillance among survivors of colorectal cancer, and substantially enhance this important aspect of survivorship care in a growing population of patients. Thus, we conducted qualitative interviews with survivors of colorectal cancer and their partners together, with the aim of describing partners’ own perspectives of their engagement in patients’ surveillance.

## 2 METHODS

### 2.1 Recruitment

Eligible participants were English‐speaking adults 21–75 years old who had undergone surgical resection of Stage III colorectal cancer within the past one to 5 years, and their partners (spouse or significant other). Participants were recruited from a large academic medical center and a mid‐sized community hospital. A random sample of 100 patients eligible for participation was selected from each institution's tumor registry. Patients were telephoned to gauge interest, confirm eligibility including presence of a partner, and obtain partner contact information. Although recruitment was not intentionally restricted to heteronormative patient‐partner dyads, the cohort included no same‐sex couples. Patient‐partner dyads were enrolled in the study and interviewed until thematic saturation was reached.

### 2.2 Interviews

From September 2016 to March 2017, we conducted in‐person, semi‐structured interviews with 20 participants (10 patients and 10 partners), who gave informed written consent prior to the interview. Patient‐partner dyads were interviewed together by two study team members and interviewed separately by one study team member. The dyadic interview lasted 60 min, and individual interviews lasted 15–20 min each. Each participant was provided a gift of $50.

### 2.3 Data collection

We developed an interview guide based on previous research on family caregiving for patients with cancer^12^, ^13^, ^14^ and the aims of the current study. Open‐ended question prompts focused on ways in which partners participated in or experienced the patient's surveillance process. We pilot‐tested and refined the interview guide by cognitively interviewing two patient‐partner dyads. All interviews were audio‐recorded and transcribed verbatim.

### 2.4 Qualitative analysis

Initially, three members of the study team independently reviewed one dyad interview, one patient interview, and one partner interview and identified emerging codes in parallel. We developed a preliminary codebook through iterative discussion‐based consensus and applied the initial codebook to all of the transcripts. Two members of the study team reduced and consolidated the codes through further discussion. We then re‐applied the final codebook to all transcripts.

We conducted a thematic analysis of utterances from partners derived from both individual and dyadic interviews in five steps: (1) Data familiarization, (2) Initial coding generation and reduction, (3) Search for themes, (4) Review and refinement of themes, (5) Theme definition and labeling.^15^ To validate our findings, we confirmed themes through triangulation between study team members and triangulation between dyadic and individual interviews, and we conducted a search for disconfirming evidence throughout all transcripts. We used qualitative data analysis software to assist in organization of all coded transcripts (VERBI Software MAXQDA 2018). This study was approved by the University of Michigan Institutional Review Board.

## 3 RESULTS

A total of 10 patients and 10 partners participated. Four patients and six partners were female; six patients and four partners were male. All patients had completed adjuvant chemotherapy. Interviews revealed nine emergent themes, which we organized into three overarching domains: (1) Psychosocial engagement, (2) Information‐seeking and dyadic communication, and (3) Instrumental engagement.

### 3.1 Domain 1: psychosocial engagement

Psychosocial engagement was defined as any partner activity that related to the psychosocial care and support of the patient, and any partner activity designed to address their own needs for support (Table 1).

**TABLE 1:** Domain: psychosocial engagement

Theme	Definition	Exemplar quotes from partners
Promoting patients’ participation in surveillance	Anything the partner does to encourage the patient to pursue surveillance care. Includes encouraging and/or nagging	*And I am very much to sticking to exactly what the doctor says and sometimes he wants to deviate because he thinks he's feeling better, he doesn't realize that you really have to take that in perspective as to when they want you to go do something else*. (Partner 3) *I nag him to go*. (Partner 2) *And we will do it, and that's it, and then we argue, and then he's… Sorry. I am the pusher. I am the pusher*. (Partner 4)
Caring and concern	Anything the partner does to support the patient emotionally	*…a big part of what we've had to do with working together on this is knowing that we'll be there for the anxiety and the worry, but, you know, we're not going to get caught up in it*. (Partner 7) *You know, if nothing else you just don't walk away from cancer, it's part of both of us, even though it happened to her, it's part of both of us…* (Partner 8)
Partner Self‐care	Anything the partner does to help care for themselves, usually as a way to make sure they can continue to support the patient through surveillance care. Includes physical or emotional self‐care	*…it is very stressful, so then I made a conscious decision to take care of myself… I’ve lost 30 some pounds in the last year and a half, started eating better, exercising more, so I have to take care of me in order to start taking care of him*. (Partner 2) *I tell my kids all the time I’m a pie, there's finite amount of me. And each of you get a slice and some of you get a little more, some of you get a little less depending on the time and what your needs are. But I have no more than that finite amount of pie*. (Partner 5)

#### 3.1.1 Theme 1. Promoting patients’ participation in surveillance

Partners described strategies they employed to urge patients to attend scheduled surveillance appointments when patients preferred not to go. Positive reinforcement strategies included encouraging patients to maintain a positive outlook, especially when patients expressed concerns about the possibility of surveillance testing revealing a recurrence of their cancer. Negative reinforcement strategies included “nagging,” expressing anger, and enlisting powerful others such as clinicians or even employers. Partners expressed the need to be vigilant for patients’ avoidance of surveillance with the excuse that they physically felt better or worse, which would cue the partners to act as authority figures. Partners indicated directly and indirectly that they found this role difficult. As one partner stated, “*…I can't tell you how many times I had to tell her I’ll take the day off to make sure you do this or I will call your [employer]… as a spouse you don't want to treat your spouse like a kid but… It's hard*” (Partner 8).

#### 3.1.2 Theme 2. Caring and concern

Partners reported providing emotional support to patients during the surveillance period. Several endorsed this as an expected and appropriate role as a partner. For example, one partner stated, “*We were together all the way…you know, you take the vows, and now I was being tested a little bit and that was my role I guess*” (Partner 4), while another stated, “*When you love somebody, you wanna take care of them, and you wanna make sure they get the best care, and make sure he takes care of himself*” (Partner 2). Partners also described “being there” as an emotional or psychological behavior, indicating that they would listen to patients’ concerns without burdening the patients with their own concerns. Some partners looked to patients to dictate the type of support that would be most helpful. As one partner stated, “*I just let her know that whatever you need, whatever it takes, that's what we're going to do. And you just let me know what that is*” (Partner 7).

#### 3.1.3 Theme 3. Partner self‐care

Noting the stressful nature of living through a spouse's cancer diagnosis, partners reported that attending to their own physical and emotional needs ensured that they could continue to remain engaged in patients’ surveillance. As one partner stated, “*…I have to take care of me in order to start taking care of him*” (Partner 2). Several partners described how this need became clear after they had become depleted. When they recognized their finite emotional resources and time, they made a conscious choice to give less to others and in some cases explicitly informed other family members that they should expect less and why. Partners reported a range of self‐care activities, including exercise, yoga, dietary modification, spending time with friends, and making time for hobbies.

### 3.2 Domain 2: information‐seeking and dyadic communication

The domain of information‐seeking and dyadic communication includes partners’ efforts to gather information and knowledge about surveillance, and to communicate about surveillance with the patient (Table 2).

**TABLE 2:** Domain: information‐seeking and dyadic communication

Theme	Definition	Exemplar quotes from partners
Gathering information	Anything the partner does to gather more information about surveillance from external sources	*I started digging through medical journals and trying to understand more about the treatments and the side effects he might have, how to help mitigate some of those things in addition to whatever we got from the cancer center*. (Partner 2) *Us getting knowledge and reading and searching for some things*. (Partner 4) *I have this pile of documentation and I’m reading online…* (Partner 8)
Staying informed about test results	Anything the partner does to keep track of the patient's surveillance test results	*His blood work… I always had to get his blood work!* (Partner 1) *I check the portal when stuff comes through… I have the [portal] app on my phone*. (Partner 2)
Communicating about surveillance	Any ways in which the partner talks about surveillance care with the patient	*We talk all the time about it when it's relevant*. (Partner 8) *All we can do is just talk about it, discuss it, grasp every situation there is*. (Partner 9) *He doesn't want to give it power and I think knowledge is power. So you have a fundamental disconnect there, right? So I as the spouse, need to get to the right level. Sometimes I need to badger, like if he says nothing I’m like that's not good enough, you gotta tell me*. (Partner 5)

#### 3.2.1 Theme 1. Gathering information

Partners reported gathering information about surveillance from external sources such as the internet and medical journals. Some partners reported a team approach to information‐seeking with the patient. One partner described using such a team approach as a concrete way to process the cancer experience: “*I mean [we] basically approached it like science or research…he's an engineer…so we approached it as a problem to deal with, rather than this ball of emotional crap*” (Partner 2). For others, gathering information was a solo effort, either as a way to help the patient or because the patient preferred not to do it. As one partner reported, “*I’m a detail person, he's not*” (Partner 5).

#### 3.2.2 Theme 2. Staying informed about test results

Partners considered medical tests important and helped to interpret or keep track of patients’ surveillance test results in a variety of ways. They described viewing test results in patients’ online portals, reviewing paper test results sent home from clinic visits, or asking about test results in person during clinic appointments. Some partners reported taking full responsibility for following test results, either because patients preferred not to know details (“*He doesn't wanna know, he just wants to know everything is good*” (Partner 5)) or because patients anticipated that partners would take responsibility (“*So [patient] just leaves it up to me, because he knows I’m going to look [at the online portal]*” (Partner 2)).

#### 3.2.3 Theme 3. Communicating about surveillance

Partners described many ways of communicating about surveillance with patients including general discussion, planning for upcoming surveillance tests and appointments, and interpreting results of surveillance tests or appointments. For partners who attended surveillance appointments with patients, communication about the appointment afterward provided an opportunity to confirm that patient and partner came away with the same information. For example, one partner stated, “*Well we talk about it before, you know, that it's important to do it. And then we talk about it afterwards like that we're agreeing what we heard when we went*” (Partner 2). For partners unable to attend surveillance appointments, communication with the patient helped the partner to stay informed and feel involved.

Beyond sharing basic details and logistics, communication about surveillance was a way that some partners engaged with patients to understand their needs and preferences, and to advocate for the patient. For example, one partner stated, “*Well I’m not the one going through the cancer, so I have to watch cues from him and then take my cues from what he needs”* (Partner 5). This partner also indicated that she sought other clues about whether the patient's clinical needs were being met and whether she may need to be more involved when she stated, “*…what he wants and what he needs aren't always the same thing… If I felt that that wasn't happening, I would be up in that appointment, I would find a way to make it work, and I’d be there saying what's going on?”* (Partner 5).

### 3.3 Domain 3: instrumental engagement

Instrumental engagement was defined as any tangible or objectively extrinsic activity of partners in clinical or domestic processes related to the patient's surveillance (Table 3).

**TABLE 3:** Domain: instrumental engagement

Theme	Definition	Exemplar quotes from partners
Scheduling and reminding patients of appointments	Anything the partner does to schedule surveillance appointments, keep track of appointments, and/or remind the patient of scheduled appointments	*Well for me, it was always making sure stuff was scheduled on the appropriate days so I could work it into my babysitting schedule, which wasn't an issue*. (Partner 1) *I check the portal to make sure, they go on my calendar. I keep track of everybody's stuff…* (Partner 2)
Attending appointments and accommodating preferences for involvement	The partner attending surveillance appointments with the patient, and/or providing the patient with transportation to/from appointments. Also, anything the partner does to accommodate the patient's preferences for how involved the partner should be in surveillance	*And I went to every doctor's appointment. And part of that was that you have another set of ears to hear everything correctly*. (Partner 1) *I just go with him, you know and sit there and just as a second ear*. (Partner 10) *Certain ones she would want me to go to. A lot of them she did not want me to go she would tell me I don't want you there*. (Partner 6)
Managing responsibilities at home	Anything the partner does to help manage domestic responsibilities so the patient can pursue surveillance care	*…the best thing that I have to do is be supportive by being able to take care of our daughter… when [patient] has to go and make sure she can get there*. (Partner 7) *Because… we got the biggest baby in the world that I got to watch out for too. He's a hundred pound chocolate lab*. (Partner 9)

#### 3.3.1 Theme 1. Scheduling and reminding patients of appointments

Most partners reported taking an active role in scheduling patients’ surveillance tests and appointments, tracking appointments on the calendar, and reminding patients of upcoming appointments. In some dyads, the partner assumed complete responsibility for these tasks both to ensure that the patient was seen and also to clear their own calendars as necessary. For example, one partner reported, “*Really I feel that that's what I need to do because I am better at keeping our schedules and keeping at it than he is*” (Partner 3). Two partners reported that patients managed scheduling without their assistance.

#### 3.3.2 Theme 2. Attending appointments and accommodating preferences for involvement

Many partners reported attending most or all surveillance appointments with the patients, and providing transportation for tests, especially colonoscopies. Partners endorsed attendance of appointments as a way to confirm the clinical information being delivered. Notably, two partners reported they desired to accompany patients to appointments but refrained because the patient preferred to attend alone. Both partners indicated a belief that it was important for the patient to feel independent or regain control of their care. One partner stated, “*She's very independent… it's a protective mode so she pretty much just handles a lot of it on her own*” (Partner 6).

#### 3.3.3 Theme 3. Managing responsibilities at home

Partners reported increasing their domestic responsibilities, such as childcare, errands, and chores, as a way to free up patients’ time and support their ability to attend surveillance appointments. As one partner stated, “*It's like okay I’ve got this scheduled and I need the house taken care of, this lawn taken care of, you got the phone with this, you know something comes up with [daughter] you're going to have to deal with it. I’m fine with all that so it's just making sure she knows that when she needs to be at those appointments that's all she's gotta worry about*.” (Partner 7).

## 4 DISCUSSION

In this qualitative study, partners of patients who previously completed curative‐intent treatment of Stage III colorectal cancer reported engagement in cancer surveillance across domains of psychosocial engagement, information‐seeking and dyadic communication, and instrumental engagement. Most partners perceived their engagement to be a positive contribution to patients’ surveillance process, helping patients manage both the emotional and logistic aspects of this part of their survivorship care. Given that over half of the 1.2 million colorectal cancer survivors in the United States currently fail to receive guideline‐concordant surveillance,^5^, ^6^ the perspectives of partners in our study provide valuable insight into their engagement in the surveillance process and suggest that partners could potentially be leveraged as a resource to help improve patients’ uptake of surveillance.

Our finding that not all partners reported concordance between their own preferences for their engagement and the patients’ preferences for level of partner engagement is an important one. Some partners preferred a greater level of engagement in surveillance but perceived that the patients themselves preferred less engagement from them. These partners interpreted the patients’ desire to navigate surveillance on their own as a way to maintain control over their health and regain autonomy. Previous studies have found that family support does not always align with patients’ desires^7^ and can be a barrier to patients’ self‐efficacy if perceived as nagging or critical.^16^, ^17^ While the partners in our study did not describe patients forgoing surveillance because of their engagement, they did report a nuanced understanding of the patients’ emotional needs and tailored their engagement to accommodate those needs. Such compromises allowed these partners to feel that their own need to stay informed about the patients’ surveillance was being met, while also respecting the patients’ need to maintain independence. Our findings highlight the sometimes complex nature of partner engagement in surveillance and underscore the fact that more instrumental engagement (for example, more attendance at clinic appointments) is not perceived as helpful by all patients.

Another important finding in our study is that in order to maintain their level of engagement in patients’ care, partners reported that they themselves require physical and emotional care and support. While the partners in our study mostly described personal behaviors, such as increasing physical activity or engaging in favorite hobbies, as a means of self‐care, they also reported seeking support from outside sources, such as friends. Though partners in our study did not specifically report a desire for increased support from the health care system, in prior work cancer survivors have expressed dissatisfaction with the limited extent to which their partners’ emotional needs were considered by the health care team.^18^ Thus, there may be opportunities within the health care system to better assess and support the needs of partners throughout the surveillance period.

### 4.1 Clinical implications

Our findings have multiple clinical implications. First, the perspectives of the partners in this study provided valuable insight into partners’ engagement in the surveillance process and suggest that clinicians should view partners as potential collaborators to help patients follow through and receive recommended surveillance. Clinicians should recognize the patient‐partner dyad as the unit of care, provide partners with the information and education needed to understand the surveillance care plan, and briefly assess partners’ needs.^19^

Second, our findings should be incorporated into interventions to improve patients’ uptake of surveillance. There is a precedent for the inclusion of partners and other family members in cancer care interventions; Several randomized controlled trials of psychoeducational and skills training interventions targeted to caregivers of patients with cancer resulted in significantly reduced caregiver burden,^20^ and a similar intervention to improve communication between patients living with cancer and their family caregivers has been piloted in a web‐based format for patients and caregivers to use together at a mutually convenient time.^21^ Informed by our findings, future research should focus on dyadic interventions to assess patients’ and partners’ personal preferences for engagement, and then, provide tailored feedback to help partners effectively engage in surveillance in ways that are perceived as helpful by patients, and to help patients appreciate and accept this engagement from their partners. Such interventions could also include education and resources for partner self‐care. By helping partners effectively engage in surveillance, dyadic interventions have the potential to improve clinical outcomes among colorectal cancer survivors by increasing their uptake of guideline‐concordant surveillance.

### 4.2 Study limitations

There were several limitations to our study that warrant mention. Although we enrolled a random sample of both male and female patients from academic and community oncology practices, the cohort lacked racial, ethnic, and geographic diversity as well as patients outside of heteronormative relationships. Our findings were reinforced by achievement of thematic saturation prior to completion of all interviews; however, it is possible that some partner perspectives were not represented. To mitigate this possibility, we conducted a systematic and iterative interpretation of the data, searched for disconfirming or contradictory examples to the identified themes, identified supporting examples for the conclusions drawn, and thoroughly reflected and discussed findings among the research team. We note that our qualitative approach is designed to elicit new knowledge and explanatory mechanisms rather than to achieve statistical representation of a population.

In summary, we have identified specific ways that partners engage in the surveillance care of survivors of colorectal cancer, as reported by partners themselves. To our knowledge, this is the first study to assess partners’ own perspectives, and thus, provides valuable insight into opportunities to better leverage partners as informal resources in cancer care, develop dyadic interventions to help partners effectively engage in ways that are helpful to patients, and, ultimately, improve receipt of guideline‐concordant surveillance among survivors of colorectal cancer.

## CONFLICT OF INTEREST

The authors report no conflicts of interest.

## ETHICAL APPROVAL STATEMENT

This study was approved by the University of Michigan Institutional Review Board (HUM00114499).

## Data Availability

The data that support the findings of this study are available from the corresponding author upon reasonable request.
